# Antithrombotic Medications and Intraocular Hemorrhage Risk in Exudative Age-Related Macular Degeneration

**DOI:** 10.1001/jamanetworkopen.2025.31366

**Published:** 2025-09-11

**Authors:** Min Seok Kim, Seonghee Nam, Jeongwoo Lee, Se Joon Woo

**Affiliations:** 1 Department of Ophthalmology, Seoul National University College of Medicine, Seoul National University Bundang Hospital, Seongnam, Korea; 2 Samil Pharm Co Ltd, Seoul, Korea

## Abstract

**Importance:**

Although the use of anticoagulants or antiplatelets is known to increase bleeding risk, there is limited evidence on whether they contribute to a higher incidence of intraocular hemorrhage in patients with age-related macular degeneration (AMD).

**Objective:**

To examine the association between anticoagulant or antiplatelet use and clinically important intraocular hemorrhage requiring vitrectomy in patients with exudative AMD.

**Design, Setting, and Participants:**

In this nationwide, population-based, retrospective cohort study using the Korean Health Insurance Review and Assessment Service database, 149 620 patients with exudative AMD older than 40 years were identified from May 1, 2014, to April 30, 2023.

**Exposures:**

Use of anticoagulants or antiplatelets.

**Main Outcomes and Measures:**

The main study outcome was clinically important intraocular hemorrhage requiring vitrectomy. The analysis used a retrospective, longitudinal cohort study design using Cox proportional hazards regression analysis and Kaplan-Meier survival analyses (exposure vs nonexposure group) and a cross-sectional case-control study design using logistic regression analysis (hemorrhage vs no hemorrhage group). All analyses were adjusted for demographics and comorbidities.

**Results:**

A total of 94 449 patients (mean [SD] age, 71.8 [9.8] years; 55 677 [59.0%] male) were included in the cohort study, and 8110 patients (mean [SD] age, 70.2 [9.6] years; 5090 [62.8%] male) were included in the case-control study. In the Cox proportional hazards regression analysis, anticoagulant or antiplatelet exposure was associated with a higher risk of intraocular hemorrhage requiring vitrectomy (adjusted hazard ratio, 1.15; 95% CI, 1.02-1.29). The incidence probability of intraocular hemorrhage requiring vitrectomy was higher in the exposure group than the nonexposure group. In the logistic analysis, the use of anticoagulants (adjusted odds ratio [aOR], 1.88; 95% CI, 1.45-2.44) or antiplatelets (aOR, 1.37; 95% CI, 1.19-1.57) was associated with intraocular hemorrhage requiring vitrectomy. The combined use of anticoagulants and antiplatelets showed the highest aOR (aOR, 2.28; 95% CI, 1.65-3.15). Higher medication adherence was also associated with a higher aOR of intraocular hemorrhage (aOR, 1.69; 95% CI, 1.45-1.97).

**Conclusions and Relevance:**

In this nationwide cohort study of patients with exudative AMD, antithrombotic medications were associated with a higher risk of intraocular hemorrhage requiring vitrectomy. These results suggest the need for proactive communication and tailored monitoring strategies to help minimize such sight-threatening complications.

## Key Points

**Question:**

Is the use of anticoagulants or antiplatelets associated with intraocular hemorrhage in age-related macular degeneration?

**Findings:**

In this cohort study of 149 620 patients with exudative age-related macular degeneration, the use of anticoagulants or antiplatelets was associated with intraocular hemorrhage requiring vitrectomy. The highest risk was observed with the combination of anticoagulants and antiplatelets, and high medication adherence was associated with increased odds of hemorrhage.

**Meaning:**

These findings suggest that patients with age-related macular degeneration should receive proactive communication and monitoring to minimize the threat of intraocular hemorrhage.

## Teaser

This cohort study of patients with exudative age-related macular degeneration assesses whether the incidence of intraocular hemorrhage was higher in patients receiving anticoagulants or antiplatelets compared with those not receiving such therapy.

## Introduction

Intraocular hemorrhage is one of the most severe complications associated with exudative age-related macular degeneration (AMD). Previous studies have reported that 6.7% to 8.0% of patients with AMD develop hemorrhagic complications, including vitreous hemorrhage and retinal hemorrhage.^1,2^ The frequency of vitreous hemorrhage is even higher in the polypoidal choroidal vasculopathy (PCV) subtype, with reported rates ranging from 12.4% to 19.9%.^3,4^ In most cases, breakthrough vitreous hemorrhage originates from large submacular hemorrhages, leading to poor long-term visual outcomes due to submacular scar formation even if vitrectomy is performed.^5^

Given the clinical importance of intraocular hemorrhage, its risk factors in patients with AMD have been investigated, including both ocular and systemic aspects. The use of anticoagulant or antiplatelet medications, which can increase systemic bleeding risk, has also been suggested as a potential risk factor for intraocular hemorrhage in AMD.^6,7,8^ However, previous studies had small sample sizes, highlighting the need for further research with large populations. Therefore, we investigated the association between systemic anticoagulant or antiplatelet use and intraocular hemorrhage requiring vitrectomy in patients with exudative AMD using large, nationwide, population-based data from Korea. The primary objective was to test the hypothesis that the incidence of intraocular hemorrhage would be higher in patients receiving anticoagulant or antiplatelet therapy compared with those not receiving such therapy. As a secondary objective, we performed a case-control analysis with logistic regression to assess whether recent anticoagulant or antiplatelet use was more frequent among patients who developed intraocular hemorrhage than among those who did not.

## Methods

The institutional review board of Seoul National University Bundang Hospital approved this study. Given the use of anonymized data and the retrospective nature of the research, the institutional review board waived the requirement for informed consent. The study adhered to the principles of the Declaration of Helsinki^9^ and followed Good Clinical Practice guidelines. We followed the Strengthening the Reporting of Observational Studies in Epidemiology (STROBE) guideline.

### Data Sources

This retrospective cohort study used data from the Health Insurance Review and Assessment (HIRA) service of Korea. HIRA is a governmental agency responsible for reviewing and evaluating most health claims in Korea, covering approximately 97% of Korean population (approximately 50 million individuals). The HIRA database offers comprehensive data, including diagnoses, procedures, prescriptions, demographics, and medical expenses. The data are available upon reasonable request and approval through the HIRA Big Data Open Portal. The analytical code used for this study was constructed based on the coding structure of the HIRA database (eMethods 1 in Supplement 1).

### Cohort Definition and Study Variables

We identified 149 620 patients with exudative AMD using the special registration code (V201) from May 1, 2014, to April 30, 2023, and extracted all of their claims during the same period. The V201 registration code is assigned after a board-certified ophthalmologist confirms exudative AMD based on optical coherence tomography and fluorescein angiography. Therefore, identifying patients with exudative AMD using the V201 code in the Korean HIRA database ensures high reliability. We then excluded patients with preexisting exudative AMD from May 1, 2014, to December 31, 2016, those younger than 40 years, and those whose first diagnosis of exudative AMD coincided with the date of their last follow-up. Race and ethnicity information was not collected because such data are not routinely recorded in the Korean national claims database. Of the 149 620 patients with exudative AMD, 94 449 patients 40 years or older who were newly diagnosed as having exudative AMD between January 1, 2017, and April 30, 2023, were included in the analysis.

The anticoagulant medications used in the analysis included warfarin, sulfomucopolysaccharide, sulodexide, and new oral anticoagulants, whereas the antiplatelet agents consisted of aspirin, P2Y12 inhibitors, selexipag, beraprost sodium, mesoglycan sodium, triflusal, sarpogrelate, cilostazol, and dipyridamole. The proportion of days covered (PDC), as used in our study, is a measure of medication adherence calculated by determining the percentage of days a patient takes the prescribed medication within a defined time frame (eg, 1 year).^10^

### Primary and Secondary Objectives

We used 2 different study designs with corresponding statistical analyses: a retrospective, longitudinal cohort study design using Cox proportional hazards regression analysis as the primary objective and a case-control study design using logistic regression analysis as the secondary objective. For the retrospective, longitudinal cohort study, we categorized patients with exudative AMD into 2 groups: exposure and nonexposure. We considered the use of anticoagulants or antiplatelets, starting 1 year before the diagnosis of exudative AMD as a time-varying covariate. The outcome event for Cox proportional hazards regression analysis was defined as the presence of a vitrectomy surgical code (S5121 for total vitrectomy or S5122 for partial vitrectomy) alongside a diagnostic code for vitreous hemorrhage (H431 or H450) or retinal hemorrhage (H356) using the date of diagnosis of exudative AMD as the index date. Kaplan-Meier survival analysis was conducted to compare the incidence probabilities of intraocular hemorrhage requiring vitrectomy based on anticoagulant or antiplatelet exposure.

**Figure 1.**
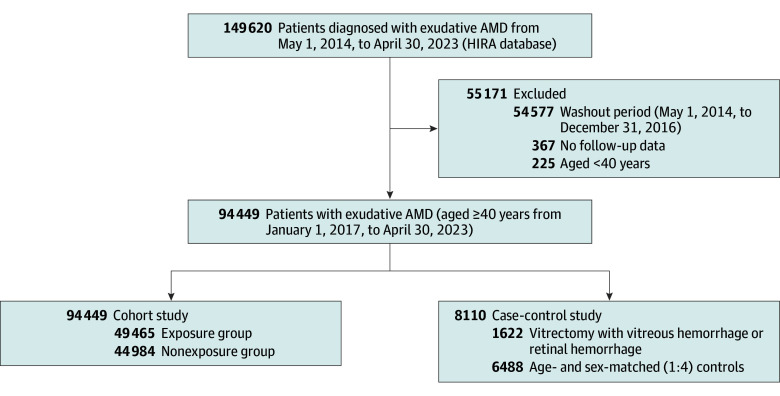
Flow Chart of Patient Participation AMD indicates age-related macular degeneration; HIRA, Health Insurance Review and Assessment.

For the case-control study, logistic regression was used to assess the association between anticoagulant or antiplatelet use and intraocular hemorrhage (vitreous hemorrhage or retinal hemorrhage) requiring vitrectomy. Patients were assigned to the case group if they had a surgical code for vitrectomy (S5121 for total vitrectomy or S5122 for partial vitrectomy) along with a diagnostic code for vitreous hemorrhage (H431 or H450) or retinal hemorrhage (H356) on the same date. Vitrectomies performed after the diagnosis of exudative AMD were included in the study. The control group included patients who had no record for vitrectomy for intraocular hemorrhage during the entire study period, matched to the case group by age and sex at a 1:4 ratio (Figure 1). Only the first vitrectomy for each patient during their study period was included in the analysis. We conducted logistic regression analysis twice, considering the type of antithrombotic medication (anticoagulants or antiplatelets) or the PDC as the independent variable. The use of anticoagulants or antiplatelets was considered during the 1-year period before the date of vitrectomy.

Both analyses were adjusted for age, sex, and other systemic comorbidities, including hypertension, diabetes, dyslipidemia, heart failure, atrial fibrillation, myocardial infarction, ischemic stroke, peripheral artery disease, and cancer. The definition of comorbidities was provided in eMethods 2 in Supplement 1. The selection of covariates was based on a priori knowledge and a directed acyclic graph constructed to reflect the hypothesized associations among use of antithrombotic drugs, intraocular hemorrhage, and potential confounders (eFigure 1 in Supplement 1). All covariates were included as categorical variables, except for age, which was treated as a continuous variable. We conducted analyses using 3 models: model 1 was unadjusted (crude); model 2 was adjusted for age and sex; and model 3 was further adjusted for comorbidities in addition to the variables in model 2.

### Statistical Analysis

Statistical analyses were performed using SAS Enterprise Guide, version 7.15 (SAS Institute Inc) and R programming, version 4.0.3 (R Foundation for Statistical Computing). SAS was used for data cleansing and preprocessing, whereas statistical analyses were conducted using both SAS and R. Visualization of the main outcomes was performed in R. Packages used in R were as follows: survival, survminer, dplyr, and MatchIt. Results were considered statistically significant at a threshold of *P* < .05, using a 2-sided hypothesis.

## Results

Of the 149 620 study patients, 94 449 (mean [SD] age, 71.8 [9.8] years; 55 677 [59.0%] male and 38 772 [41.1%] female) were included in the cohort study, and 8110 patients (mean [SD] age, 70.2 [9.6] years; 5090 [62.8%] male and 3020 [37.2%] female) were included in the case-control study. There were no missing values included in this analysis. No patients were lost to follow-up because censoring was applied at each patient’s last claim date.

### Primary Objective

**Figure 2.**
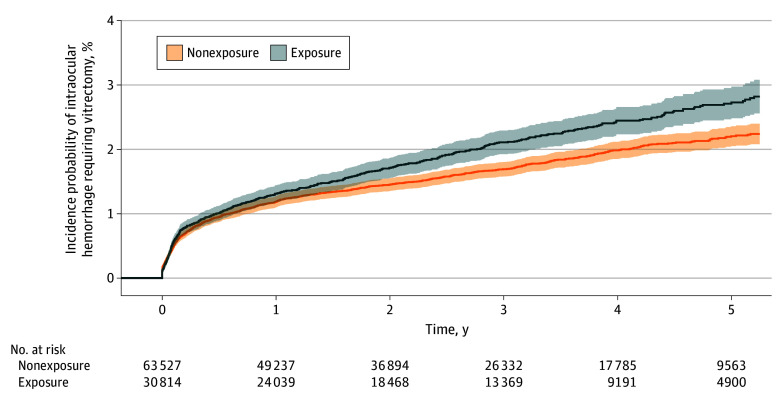
Kaplan-Meier Curves Illustrating the Incidence Probability of Intraocular Hemorrhage Requiring Vitrectomy in Patients With Exudative Age-Related Macular Degeneration Exposure was defined as the use of anticoagulants or antiplatelets starting within 1 year before the diagnosis of exudative age-related macular degeneration, treated as a time-varying covariate; nonexposure referred to patients without such medication use. Shaded areas indicate 95% CIs.

In the retrospective, longitudinal cohort study, a total of 49 465 anticoagulant or antiplatelet exposure cases and 44 984 nonexposure cases were included. Compared with the nonexposure group, the exposure group included more male patients (29 400 [59.4%] vs 26 277 [58.4%], *P* = .001), were older (73.9 [8.9] vs 69.5 [10.1] years, *P* < .001), and had a higher prevalence of comorbidities (Table 1). Among the 94 449 total patients, intraocular hemorrhage requiring vitrectomy occurred in 1622 patients (1.7%). The proportional hazards assumption was assessed using Schoenfeld residuals. All variables satisfied the assumption except for dyslipidemia and myocardial infarction (eTable 1 in Supplement 1). In the Cox proportional hazards regression analysis, the use of anticoagulants or antiplatelets was associated with a higher risk of intraocular hemorrhage requiring vitrectomy in model 3 (adjusted hazard ratio, 1.15; 95% CI, 1.02-1.29; *P* = .03) (Table 2). Male sex, younger age, diabetes, and atrial fibrillation were also associated with a higher risk of intraocular hemorrhage requiring vitrectomy. The incidence probability of intraocular hemorrhage requiring vitrectomy was higher among the exposure group than the nonexposure group (Figure 2).

**Table 1.** Demographic Characteristics of the Study Patients

Characteristic	No. (%) of patients^a^							
	Cohort study				Case-control study			
	Total (N = 94 449)	Exposure (n = 49 465)	Nonexposure (n = 44 984)	*P* value	Total (N = 8110)	Case (n = 1622)	Control (n = 6488)	*P* value
Sex								
Male	55 677 (59.0)	29 400 (59.4)	26 277 (58.4)	.001	5090 (62.8)	1016 (62.6)	4074 (62.8)	.86
Female	38 772 (41.1)	20 065 (40.6)	18 707 (41.6)		3020 (37.2)	606 (37.4)	2414 (37.2)	
Age, mean (SD), y	71.8 (9.8)	73.9 (8.9)	69.5 (10.1)	<.001	70.2 (9.6)	70.5 (9.6)	70.1 (9.5)	.01
Age group, y								
40-49	1163 (1.2)	283 (0.6)	880 (2.0)	<.001	118 (1.5)	22 (1.4)	96 (1.5)	.03
50-59	9941 (10.5)	2989 (6.0)	6952 (15.5)		1123 (13.8)	211 (13.0)	912 (14.1)	
60-69	25 548 (27.1)	11 297 (22.8)	14 251 (31.7)		2359 (29.1)	473 (29.2)	1886 (29.1)	
70-79	35 612 (37.7)	20 869 (42.2)	14 743 (32.8)		3077 (37.9)	597 (36.8)	2480 (38.2)	
80-89	20 531 (21.7)	13 008 (26.3)	7523 (16.7)		1362 (16.8)	305 (18.8)	1057 (16.3)	
≥90	1654 (2.0)	1019 (2.1)	635 (1.4)		71 (0.9)	14 (0.9)	57 (0.9)	
Antithrombotic drug								
None	NA	NA	NA	NA	4981 (61.4)	877 (54.1)	4104 (63.3)	<.001
Anticoagulant only	NA	NA	NA		398 (4.9)	111 (6.8)	287 (4.4)	
Antiplatelet only	NA	NA	NA		2526 (31.1)	568 (35)	1958 (30.2)	
Anticoagulant and antiplatelet	NA	NA	NA		205 (2.5)	66 (4.1)	139 (2.1)	
PDC of antithrombotic drug								
0	NA	NA	NA	NA	4981 (61.4)	877 (54.1)	4104 (63.3)	<.001
0-0.8	NA	NA	NA		1387 (17.1)	290 (17.9)	1097 (16.9)	
≥0.8	NA	NA	NA		1742 (21.5)	455 (28.1)	1287 (19.8)	
Comorbidities								
Hypertension	57 469 (60.9)	37 330 (75.5)	20 139 (44.8)	<.001	4857 (59.9)	1008 (62.2)	3849 (59.3)	.03
Diabetes	23 904 (25.3)	16 563 (33.5)	7341 (16.3)	<.001	2102 (25.9)	483 (29.8)	1619 (25.0)	<.001
Dyslipidemia	46 191 (48.9)	30 661 (62.0)	15530 (34.5)	<.001	3940 (48.6)	808 (49.8)	3132 (48.3)	.26
Heart failure	6851 (7.3)	5718 (11.6)	1133 (2.5)	<.001	515 (6.4)	125 (7.7)	390 (6.0)	.01
Atrial fibrillation	3905 (4.1)	3738 (7.6)	167 (0.4)	<.001	339 (4.2)	83 (5.1)	256 (4.0)	.04
Myocardial infarction	1456 (1.5)	1357 (2.7)	99 (0.2)	<.001	124 (1.5)	30 (1.9)	94 (1.5)	.24
Ischemic stroke	6978 (7.4)	6551 (13.2)	427 (1.0)	<.001	550 (6.8)	120 (7.4)	430 (6.6)	.24
Peripheral artery disease	21 359 (22.6)	16 775 (33.9)	4584 (10.2)	<.001	1791 (22.1)	374 (23.1)	1417 (21.8)	.29
Cancer	9742 (10.3)	5419 (11.0)	4323 (9.6)	<.001	837 (10.3)	154 (9.5)	683 (10.5)	.22

Abbreviations: NA, not applicable; PDC, proportion of days covered.

^a^ Unless otherwise indicated.

**Table 2.** Cox Proportional Hazards Regression Analysis Results for Intraocular Hemorrhage Requiring Vitrectomy

Characteristic	Model 1		Model 2		Model 3	
	Crude HR (95% CI)	*P* value	Adjusted HR (95% CI)	*P* value	Adjusted HR (95% CI)	*P* value
Antithrombotic drug						
Nonexposure	1.0 [Reference]	NA	1.0 [Reference]	NA	1.0 [Reference]	NA
Exposure	1.19 (1.08-1.32)	.001	1.25 (1.13-1.39)	<.001	1.15 (1.02-1.29)	.03
Sex						
Male	NA	NA	1.0 [Reference]	NA	1.0 [Reference]	NA
Female	NA		0.88 (0.80-0.98)	.02	0.89 (0.80-0.99)	.03
Age	NA	NA	0.99 (0.99-0.99)	<.001	0.99 (0.98-0.99)	<.001
Comorbidities						
Hypertension	NA	NA	NA	NA	1.05 (0.93-1.17)	.45
Diabetes	NA		NA		1.26 (1.12-1.41)	<.001
Dyslipidemia	NA		NA		1.00 (0.89-1.11)	.95
Heart failure	NA		NA		1.05 (0.86-1.27)	.66
Atrial fibrillation	NA		NA		1.28 (1.01-1.62)	.04
Myocardial infarction	NA		NA		1.13 (0.78-1.63)	.52
Ischemic stroke	NA		NA		0.95 (0.78-1.15)	.59
Peripheral artery disease	NA		NA		1.02 (0.90-1.15)	.78
Cancer	NA		NA		0.94 (0.79-1.11)	.44

Abbreviations: HR, hazard ratio; NA, not applicable.

### Secondary Objective

In the case-control study, a total of 1622 cases and 6488 controls were included. There were no significant differences in sex between the case and control groups (1016 [62.6%] and 4074 [62.8%] male, respectively; *P* = .86). Case patients who underwent vitrectomy for intraocular hemorrhage were more likely to have been prescribed anticoagulants or antiplatelets (745 [45.9%] vs 2384 [36.7%], *P* < .001) and exhibited a higher PDC (*P* < .001) (Table 1). Intraocular hemorrhage requiring vitrectomy was associated with the use of anticoagulants (adjusted odds ratio [aOR], 1.88; 95% CI, 1.45-2.44; *P* < .001) and antiplatelets (aOR, 1.37; 95% CI, 1.19-1.57; *P* < .001) in the multivariate logistic regression analysis. The combined use of both anticoagulants and antiplatelets demonstrated a higher aOR (aOR, 2.28; 95% CI, 1.65-3.15; *P* < .001) compared with single use (Table 3; eFigure 2 in Supplement 1). When considering the PDC as an independent variable, the ranges of 0 to 0.8 (aOR, 1.27; 95% CI, 1.08-1.49; *P* = .003) and 0.8 or greater (aOR, 1.69; 95% CI, 1.45-1.97; *P* < .001) were associated with intraocular hemorrhage requiring vitrectomy (Table 3; eFigure 3 in Supplement 1). Additionally, age (aOR, 1.01; 95% CI, 1.00-1.03; *P* = .02) and diabetes (aOR, 1.19; 95% CI, 1.04-1.36; *P* = .01) were identified as factors associated with a higher risk of intraocular hemorrhage requiring vitrectomy in model 3, alongside the use of anticoagulants or antiplatelets. In both the cohort (χ^2^ = 66.1, *df* = 12, *P* < .001) and case-control analyses (type of antithrombotic medication as a covariate, χ^2^ = 80.5, *df* = 14, *P* < .001; PDC as a covariate, χ^2^ = 78.2, *df* = 13, *P* < .001), likelihood ratio tests showed statistically significant improvement in model fit, supporting the adequacy of the multivariable models.

**Table 3.** Logistic Regression Analysis Results for Intraocular Hemorrhage Requiring Vitrectomy by Type and PDC of Antithrombotic Medication

Independent variable	Model 1		Model 2		Model 3		Model 1		Model 2		Model 3	
	Crude OR (95% CI)	*P* value	aOR (95% CI)	*P* value	aOR (95% CI)	*P* value	Crude OR (95% CI)	*P* value	aOR (95% CI)	*P* value	aOR (95% CI)	*P* value
None	1.0 [Reference]	NA	1.0 [Reference]	NA	1.0 [Reference]	NA	NA	NA	NA	NA	NA	NA
Anticoagulant only	1.83 (1.45-2.31)	<.001	1.80 (1.43-2.28)	<.001	1.88 (1.45-2.44)	<.001	NA	NA	NA	NA	NA	NA
Antiplatelet only	1.38 (1.22-1.55)	<.001	1.36 (1.21-1.54)	<.001	1.37 (1.19-1.57)	<.001	NA	NA	NA	NA	NA	NA
Anticoagulant and antiplatelet	2.24 (1.65-3.03)	<.001	2.20 (1.63-2.99)	<.001	2.28 (1.65-3.15)	<.001	NA	NA	NA	NA	NA	NA
PDC												
0	NA	NA	NA	NA	NA	NA	1.0 [Reference]	NA	1.0 [Reference]	NA	1.0 [Reference]	NA
0 -<0.8	NA	NA	NA	NA	NA	NA	1.25 (1.08-1.46)	.003	1.25 (1.07-1.45)	.004	1.27 (1.08-1.49)	.003
≥0.8	NA	NA	NA	NA	NA	NA	1.68 (1.47-1.92)	<.001	1.66 (1.45-1.89)	<.001	1.69 (1.45-1.97)	<.001
Sex (female)	NA	NA	1.18 (0.97-1.44)	.11	1.20 (0.98-1.46)	.08	NA	NA	1.17 (0.96-1.43)	.12	1.20 (0.98-1.46)	.08
Age	NA	NA	1.01 (1.00-1.03)	.02	1.01 (1.00-1.03)	.02	NA	NA	1.01 (1.00-1.03)	.02	1.01 (1.00-1.03)	.02
Comorbidities												
Hypertension	NA	NA	NA	NA	1.00 (0.88-1.13)	.95	NA	NA	NA	NA	0.97 (0.85-1.11)	.67
Diabetes	NA	NA	NA	NA	1.21 (1.06-1.38)	.004	NA	NA	NA	NA	1.19 (1.04-1.36)	.01
Dyslipidemia	NA	NA	NA	NA	0.90 (0.79-1.02)	.10	NA	NA	NA	NA	0.88 (0.78-1.00)	.05
Heart failure	NA	NA	NA	NA	1.10 (0.88-1.39)	.40	NA	NA	NA	NA	1.11 (0.88-1.39)	.38
Atrial fibrillation	NA	NA	NA	NA	0.88 (0.65-1.18)	.38	NA	NA	NA	NA	1.03 (0.79-1.36)	.81
Myocardial infarction	NA	NA	NA	NA	1.07 (0.70-1.63)	.77	NA	NA	NA	NA	1.04 (0.68-1.60)	.85
Ischemic stroke	NA	NA	NA	NA	0.95 (0.76-1.18)	.64	NA	NA	NA	NA	0.93 (0.74-1.16)	.51
Peripheral artery disease	NA	NA	NA	NA	0.97 (0.84-1.11)	.62	NA	NA	NA	NA	0.95 (0.83-1.09)	.48
Cancer	NA	NA	NA	NA	0.84 (0.70-1.02)	.07	NA	NA	NA	NA	0.86 (0.71-1.03)	.11

Abbreviations: aOR, adjusted odds ratio; NA, not applicable.

## Discussion

In this large, population-based study, we demonstrated that the use of antithrombotic medications, including anticoagulants and antiplatelets, was associated with a higher risk of intraocular hemorrhage requiring vitrectomy in patients with exudative AMD. This association was consistently observed across both a retrospective, longitudinal cohort study and a case-control study, strengthening the robustness of our findings.

When intraocular hemorrhage occurs in exudative AMD, it is usually associated with subretinal hemorrhage originating from choroidal neovascularization. Large subretinal hemorrhages can lead to photoreceptor damage and the formation of a disciform scar through mechanisms such as iron toxicity, blockage of nutrient diffusion, and clot retraction.^6^ As a result, even if intraocular hemorrhage is removed via vitrectomy, the visual prognosis is generally poor.^11,12,13^ Therefore, identifying the risk factors for intraocular hemorrhage in exudative AMD is a clinically important step.

Previous reports have identified that onset at an older age, retinal pigment epithelium detachment, prior photodynamic therapy, larger subretinal hemorrhage, higher white blood cell count, higher aspartate aminotransferase to alanine aminotransferase ratio, longer activated partial thromboplastin time, PCV subtype, intravitreal injections, and antithrombotic medications as risk factors for intraocular hemorrhage in AMD.^3,6,7,8,14,15,16,17^ Previous evidence^6,7,8^ suggests that patients with AMD who are receiving anticoagulant or antiplatelet therapy have an increased risk of hemorrhagic complications. In a retrospective, single-center study involving 195 eyes from 195 patients with neovascular AMD, the cumulative incidence of intraocular hemorrhage was higher among patients taking daily oral anticoagulant or antiplatelet medications compared with nonusers (63.5% vs 29.2%).^6^ Another study suggests that anticoagulant or antiplatelet medications are strongly linked to the development of large subretinal hemorrhages in 71 consecutive patients with AMD.^7^ Shin et al^8^ observed that a higher proportion of patients with breakthrough vitreous hemorrhage in AMD combined with submacular hemorrhage were taking anticoagulants (14 of 31 [45.2%]) compared with the control group (16 of 87 [18.4%]). These studies^6,7,8^ evaluated antithrombotic medication use in a cross-sectional manner at the time of hemorrhage, which did not account for the duration of medication use in the analysis. Additionally, other studies^14,15,16^ have reached conflicting conclusions, stating that antithrombotic medications are not correlated with intraocular hemorrhage in patients with AMD, without providing an explanation for these findings. One possible reason for these conflicting results is the small number of patients included in previous studies.^6,7,8,14,16^

Our study included a large number of patients from the nationwide population data, enhancing the reliability of the results. Notably, by incorporating the PDC during a 1-year period as a variable, we observed a higher risk in patients who had prolonged anticoagulant or antiplatelet use. Interestingly, the risk of intraocular hemorrhage requiring vitrectomy was higher in patients who used both anticoagulants and antiplatelets compared with those who used either medication alone. The combination of anticoagulant and antiplatelet therapy also raises the risk of systemic bleeding in patients with acute coronary syndromes and atrial fibrillation.^18,19^

It is well established that the risk of major or minor systemic hemorrhagic complications, such as intracranial hemorrhage, gastrointestinal hemorrhage, hematemesis, and hemarthrosis, is closely related to anticoagulant or antiplatelet use.^20,21^ However, the pharmacokinetics and pharmacodynamics of anticoagulants or antiplatelets within the eye are not well understood. Superstein et al^22^ reported that 3% (4 of 126) of patients taking warfarin, who did not have any preexisting ocular conditions, had incidental retinal hemorrhage during a dilated eye examination. A study using Taiwan’s clams data found that the use of aspirin, warfarin, or clopidogrel was a significant factor for vitreous hemorrhage, with a hazard ratio of 2.2, although it did not adjust for underlying diseases.^23^ Based on evidence from previous clinical studies,^22,23^ the use of anticoagulants or antiplatelets appears to increase the risk of intraocular hemorrhage, and this risk is likely to be even higher in cases where AMD with choroidal neovascularization is present.

In this study, we selected vitrectomy for intraocular hemorrhage as the outcome of the analysis to enhance specificity and clinical relevance. Although diagnostic codes for retinal or vitreous hemorrhage may be prone to misclassification or overcoding, vitrectomy indicates a hemorrhage enough to require surgical intervention, providing a more reliable and meaningful endpoint.

We tested whether each covariate acts as a modifier of association between antithrombotic use and intraocular hemorrhage by including interaction terms and stratified analyses in the time-varying Cox proportional hazards regression model. However, no covariate demonstrated statistically or clinically meaningful evidence of being a modifier of the association in this analysis (eTable 2 in Supplement 1). Therefore, we concluded that there is no covariate with sufficient reasons to be considered as an effect modifier.

There may be a concern that the systemic comorbidities requiring anticoagulant or antiplatelet use, such as cardiovascular disease, peripheral arterial disease, deep venous thrombosis, or elevated serum cholesterol level, could have influenced the risk of intraocular hemorrhage.^6^ However, we adjusted for 9 systemic comorbidities and still found an association between anticoagulant or antiplatelet use and intraocular hemorrhage. This finding suggests that the association between anticoagulant or antiplatelet use and intraocular hemorrhage exists independently of systemic comorbidities.

Both Cox proportional hazards regression and logistic regression analyses identified diabetes as another risk factor for intraocular hemorrhage requiring vitrectomy. This finding is likely because diabetic retinopathy contributed to an increased risk of intraocular hemorrhage in patients with exudative AMD, but additional study is needed to reveal the independent association. Although age was associated with a slightly lower risk in the Cox proportional hazards regression and a slightly higher risk in the logistic regression, both estimates (adjusted HR and aOR) were close to 1, suggesting limited clinical relevance.

Although anti–vascular endothelial growth factor (anti-VEGF) therapy is the mainstay treatment for AMD, breakthrough vitreous hemorrhage may occur after intravitreal anti-VEGF injections, particularly in patients with AMD and submacular hemorrhages.^8^ Therefore, including anti-VEGF use as a covariate in the analysis would be appropriate. However, due to the diversity in anti-VEGF agents and treatment patterns, this variable was not incorporated into the current study. A separate study comparing the risk of intraocular hemorrhage according to the type of anti-VEGF agent is currently underway and will be reported in the future.

### Limitations

There are limitations to our study. Owing to the restricted scope of claims data, more detailed information necessary for sharpening the results was unavailable. First, we were unable to categorize and analyze exudative AMD by subtypes such as PCV, and changes in prothrombin time and international normalized ratio after anticoagulant or antiplatelet use were also unidentifiable. Second, although the regression models in our study were a good fit and the assumption for the Cox proportional hazards regression model was met according to statistical tests, there is a possibility of model fit limited by residual or latent confounders, such as socioeconomic factors and lifestyle factors, directly related to the outcome.

## Conclusions

Antithrombotic medications were associated with intraocular hemorrhage in Korean patients with exudative AMD. For patients with exudative AMD, it is crucial for ophthalmologists and internists to discuss whether the benefits of anticoagulant or antiplatelet use outweigh the potential adverse effects. If patients with exudative AMD are taking anticoagulants or antiplatelets, they should be informed about the risk of intraocular hemorrhage, and regular monitoring for not only exudative AMD but also intraocular hemorrhage should be conducted.

## Notes

Supplement 1. **eMethods 1.** Analytical code used for this study **eMethods 2.** The definition of comorbidities **eFigure 1.** Directed acyclic graph showing assumed relationship between drug exposure, intraocular hemorrhage, and covariates included in the model **eFigure 2.** Forest plot illustrating the results of the logistic regression analysis, with the type of antithrombotic medication as the independent variable **eFigure 3.** Forest plot illustrating the results of the logistic regression analysis, with the proportion of days covered (PDC) as the independent variable **eTable 1.** Schoenfeld residuals test results for proportional hazards assumption **eTable 2.** Stratified analysis of the association between antithrombotic use and intraocular hemorrhage

Supplement 2.
Data Sharing Statement
