# Supplementary material for: Antithrombotic Medications and Intraocular Hemorrhage Risk in Exudative Age-Related Macular Degeneration
Source: JAMA Netw Open. 2025 Sep 11;8(9):e2531366. doi: 10.1001/jamanetworkopen.2025.31366 (PMC12426794; doi:10.1001/jamanetworkopen.2025.31366)
Supplement: Supplement 1. — eMethods 1. Analytical code used for this study eMethods 2. The definition of comorbidities eFigure 1. Directed acyclic graph showing assumed relationship between drug exposure, intraocular hemorrhage, and covariates included in the model eFigure 2. Forest plot illustrating the results of the logistic regression analysis, with the type of antithrombotic medication as the independent variable eFigure 3. Forest plot illustrating the results of the logistic regression analysis, with the proportion of days covered (PDC) as the independent variable eTable 1. Schoenfeld residuals test results for proportional hazards assumption eTable 2. Stratified analysis of the association between antithrombotic use and intraocular hemorrhage [file jamanetwopen-e2531366-s001.pdf]

Supplemental Online Content

Kim MS, Nam S, Lee J, Woo SJ. Antithrombotic medications and risk of intraocular hemorrhage in exudative age-related macular degeneration. *JAMA Netw Open*. 2025;8(9):e2531366. doi:10.1001/jamanetworkopen.2025.31366

eMethods 1. Analytical code used for this study

eMethods 2. The definition of comorbidities

eFigure 1. Directed acyclic graph showing assumed relationship between drug exposure, intraocular hemorrhage, and covariates included in the model

eFigure 2. Forest plot illustrating the results of the logistic regression analysis, with the type of antithrombotic medication as the independent variable

eFigure 3. Forest plot illustrating the results of the logistic regression analysis, with the proportion of days covered (PDC) as the independent variable

eTable 1. Schoenfeld residuals test results for proportional hazards assumption

eTable 2. Stratified analysis of the association between antithrombotic use and intraocular hemorrhage

This supplemental material has been provided by the authors to give readers additional information about their work.

© 2025 Kim MS et al. *JAMA Network Open.*

eMethods 1. Analytical code used for this study

Matching for case-control study (R)

```
library(MatchIt)
library(dplyr)

casecontrol <- read.csv("/vol/userdata2/sta_room045/R/casecontrol.csv")
match_fit_vh <- matchit(vh ~ SEX_TP_CD + ageg,
                        method='nearest',
                        caliper=0.2,
                        ratio=4, data=casecontrol)

matched_dat_vh <- match.data(match_fit_vh)
matched_dat_vh$subclass <-
  vapply(rownames(matched_dat_vh),
         function(x){
           out <-
             which(rownames(match_fit_vh$match.matrix) == x |
                     apply(match_fit_vh$match.matrix, 1,
                           function(y) x %in% y))
           if (length(out) == 0) out <-
               NA_integer_
           out
         }, integer(1L))
write.csv(matched_dat_vh, file="psm4.csv")
```

Conditional logistic regression (SAS)

```
data work.casecontrol2;
set work.casecontrol;
time=1;
run;
%macro clogi(var=);
/*Model 1 (crude)*/
proc phreg data=work.casecontrol2;
```

© 2025 Kim MS et al. *JAMA Network Open.*

```
class &var.(ref='0') / param=ref;
model time*vh(0) = &var / ties=discrete rl;
strata subclass;
run;
/*Model 2 (sex, age adjusted)*/
proc phreg data=work.casecontrol2;
class &var.(ref='0') sex_tp_cd(ref='1') / param=ref;
model time*vh(0) = &var sex_tp_cd pat_age / ties=discrete rl;
strata subclass;
run;
/*Model 3 (sex, age, comorbidities adjusted)*/
proc phreg data=work.casecontrol2;
class &var.(ref='0') sex_tp_cd(ref='1') htn(ref='0') dm(ref='0') dyslipid(ref='0') hf(ref='0') af(ref='0') mi(ref='0') stroke(ref='0') pad(ref='0') cancer(ref='0') / param=ref;
model time*vh(0) = &var sex_tp_cd pat_age htn dm dyslipid hf af mi stroke pad cancer / ties=discrete rl;
strata subclass;
run;
%mend;
%clogi (var=pdc_g) %clogi (var=drug)
```

Time-varying Cox proportional hazards model (SAS)

```
/*Model 1 (crude)*/
proc phreg data=work.tdc_cohort_countp;
class drug(ref='0') / param=ref;
model (start, stop)*vh(0) = drug / ties=breslow rl;
run;
/*Model 2 (sex, age adjusted)*/
proc phreg data=work.tdc_cohort_countp;
class drug(ref='0') sex_tp_cd(ref='1') / param=ref;
model (start, stop)*vh(0) = sex_tp_cd pat_age drug / ties=breslow rl;
run;
/*Model 3 (sex, age, comorbidities adjusted)*/
proc phreg data=work.tdc_cohort_countp;
```

© 2025 Kim MS et al. *JAMA Network Open.*

```
class drug(ref='0') sex_tp_cd(ref='1') htn(ref='0') dm(ref='0') dyslipid(ref='0') hf(ref='0') af(ref='0') mi(ref='0') stroke(ref='0') pad(ref='0') cancer(ref='0') / param=ref;

model (start, stop)*vh(0) = sex_tp_cd pat_age htn dm dyslipid hf af mi stroke pad cancer drug / ties=breslow rl;

run;
```

Survival curve (R)

```
tdc <- read.csv("tdc_cohort.csv")

surv_tdc <- survfit(Surv(start, stop, vh==1)~drug+cluster(JID), data=tdc)

km <- ggsurvplot(surv_tdc, risk.table=T, risk.table.height=0.2, risk.table.fontsize = 4,

legend.labs = c("Non-exposure", "Exposure"),

                    legend.title = "",

                    conf.int=TRUE,

pval = F,

ylab="Incidence probability of |n intraocular hemorrhage requiring vitrectomy (%)",

xlab="Time (years)",

                    surv.scale = "percent",

                    ggtheme = theme_classic(), tables.theme = theme_cleantable(),

                    censor=F,

                    fun='event',

                    xscale = 365.25,

break.x.by=365.25*1

)

km$plot <- km$plot+

  theme(legend.position=c(0.18, 0.98)) +

  guides(col=guide_legend(ncol=2)) +

  scale_y_continuous(breaks = seq(0, 0.03, by=0.01), labels=c('0', '1', '2', '3'))

km
```

© 2025 Kim MS et al. *JAMA Network Open.*

eMethods 2. The definition of comorbidities

The comorbidities were identified by ICD-10 codes and medication prescription records: for hypertension, I10-I13, I15 and minimum one prescription of anti-hypertensive drug(thiazirde, loop diuretics, aldosterone antagonist, alpha-/beta-blocker, calcium-channel blocker, angiotensin-converting enzyme inhibitor, angiotensin II receptor blocker); for diabetes mellitus, E11-E14 and minimum one prescription of anti-diabetic drugs(sulfonylureas, metformin, meglitinides, thiazolidinediones, dipeptidyl peptidase-4 inhibitors, α-glucosidase inhibitors, and insulin); for dyslipidemia, E78 and minimum 1 prescription of anti-dyslipidemia drug(statin, fenofibrate, omega-3); for heart failure, I50; for atrial fibrillation, I48; for myocardial infarction, I21 and I22; for ischemic stroke, I63 and I64; for peripheral artery disease, I70 and I73; for cancer, C00-C97.

© 2025 Kim MS et al. *JAMA Network Open.*

eFigure 1. Directed acyclic graph showing assumed relationship between drug exposure, intraocular hemorrhage, and covariates included in the model.

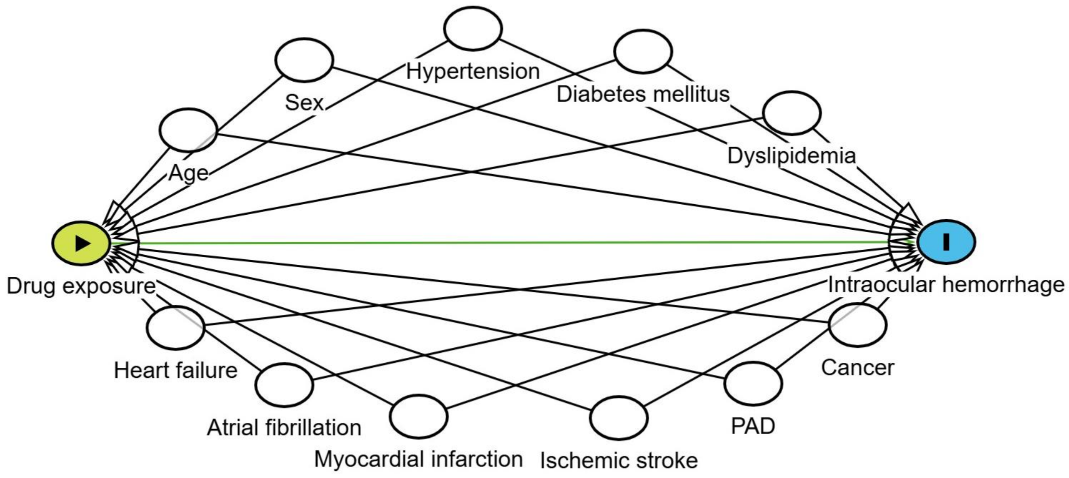

© 2025 Kim MS et al. *JAMA Network Open.*

eFigure 2. Forest plot illustrating the results of the logistic regression analysis, with the type of antithrombotic medication as the independent variable. AC = anticoagulant, AP = antiplatelet, PDC = proportion of days covered

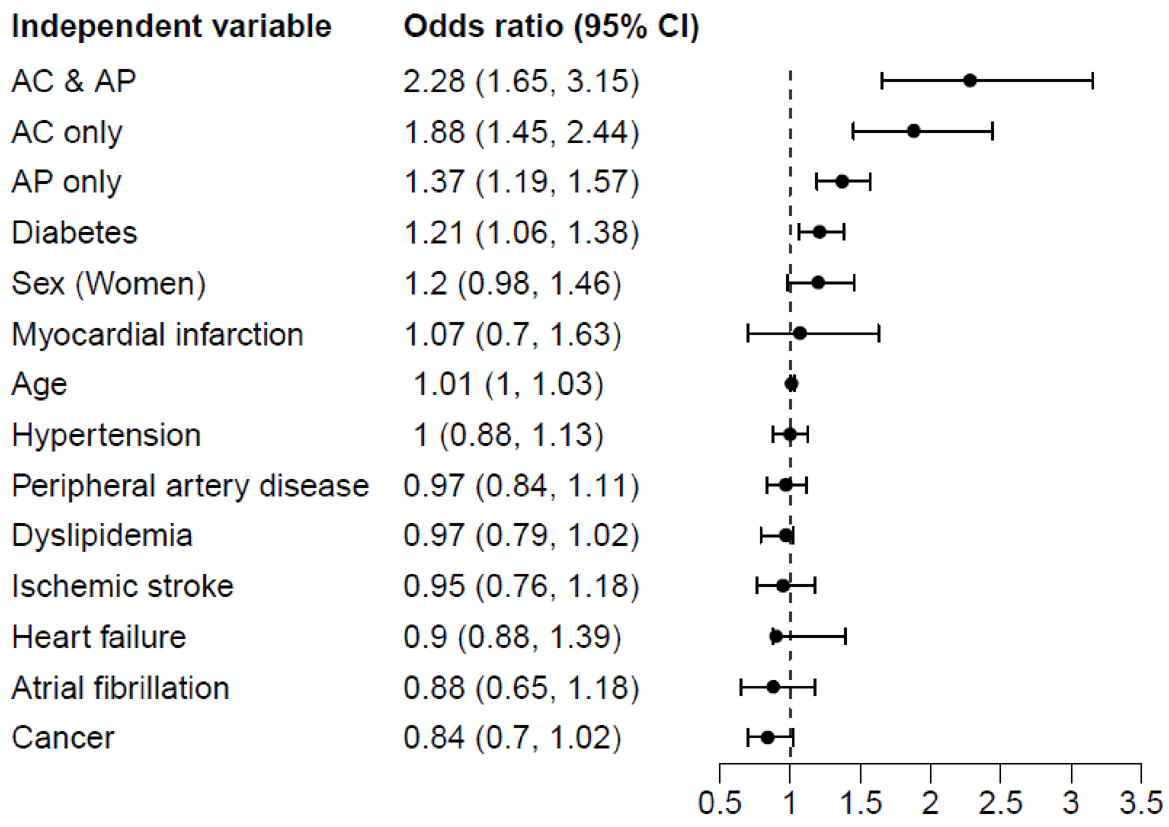

© 2025 Kim MS et al. *JAMA Network Open.*

eFigure 3. Forest plot illustrating the results of the logistic regression analysis, with the proportion of days covered (PDC) as the independent variable. AC = anticoagulant, AP = antiplatelet

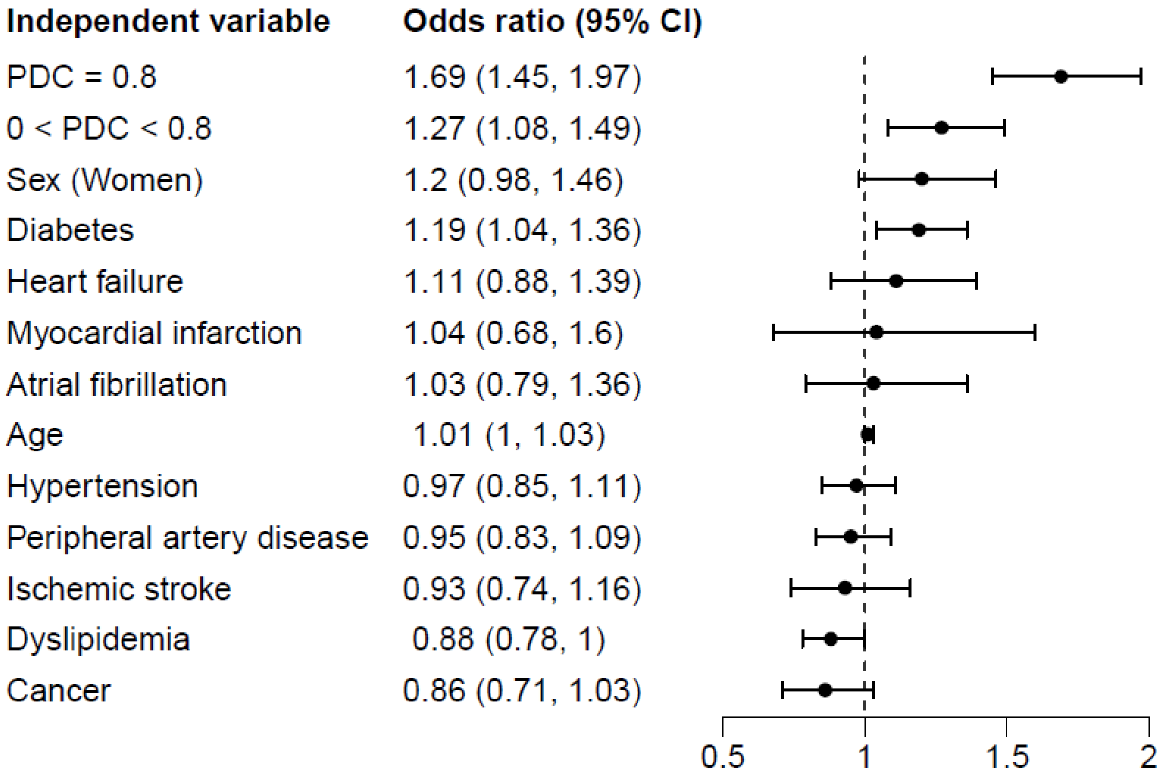

© 2025 Kim MS et al. *JAMA Network Open.*

eTable 1. Schoenfeld Residuals Test Results for Proportional Hazards Assumption

	Model 1		Model 2		Model 3	
	chisq	*P*	chisq	*P*	chisq	*P*
Antithrombotic drug exposure	3.09	.08	2.72	.10	0.33	.56
Sex						
Men	NA		reference		reference	
Women	NA		0.77	.38	1.27	.26
Age	NA		0.00	.95	0.01	.91
Comorbidities						
Hypertension	NA		NA		0.22	.64
Diabetes	NA		NA		1.34	.25
Dyslipidemia	NA		NA		11.51	.001
Heart failure	NA		NA		0.08	.77
Atrial fibrillation	NA		NA		0.02	.9
Myocardial infarction	NA		NA		5.73	.02
Ischemic stroke	NA		NA		0.16	.69
Peripheral artery disease	NA		NA		0.05	.82
Cancer	NA		NA		0.17	.68

© 2025 Kim MS et al. *JAMA Network Open.*

eTable 2. Stratified analysis of the association between antithrombotic use and intraocular hemorrhage

	Antithrombotic use		
	adjusted hazard ratio	95% confidence interval	*P*
Sex			
Men	1.14	0.98, 1.33	.08
Women	1.15	0.95, 1.4	.16
Age group			
40-49	0.95	0.25, 3.53	.93
50-59	1.33	0.91, 1.95	.15
60-69	1.20	0.95, 1.50	.12
70-79	1.10	0.91, 1.33	.34
80-89	1.06	0.81, 1.37	.69
≥90	2.50	0.73, 8.51	.14
Hypertension			
No	1.11	0.86, 1.43	.42
Yes	1.16	1.01, 1.33	.03
Diabetes			
No	1.16	0.99, 1.35	.06
Yes	1.12	0.92, 1.36	.26
Dyslipidemia			
No	1.17	0.96, 1.42	.12
Yes	1.14	0.98, 1.33	.10
Heart failure			
No	1.18	1.04, 1.33	.01
Yes	0.84	0.56, 1.27	.41
Atrial fibrillation			
No	1.15	1.02, 1.3	.03
Yes	0.93	0.53, 1.61	.79
Myocardial infarction			
No	1.13	1.01, 1.28	.04
Yes	2.78	0.63, 12.37	.18
Ischemic stroke			
No	1.15	1.01, 1.3	.03
Yes	1.07	0.66, 1.72	.80
Peripheral artery disease			
No	1.15	1, 1.33	.06
Yes	1.12	0.9, 1.4	.32
Cancer			
No	1.16	1.02, 1.31	.02
Yes	1.06	0.73, 1.54	.75

© 2025 Kim MS et al. *JAMA Network Open.*
