# Supplementary material for: Antithrombotic Medications and Intraocular Hemorrhage Risk in Exudative Age-Related Macular Degeneration
Source: JAMA Netw Open. 2025 Sep 11;8(9):e2531366. doi: 10.1001/jamanetworkopen.2025.31366 (PMC12426794; doi:10.1001/jamanetworkopen.2025.31366)
Supplement: Supplement 2. — Data Sharing Statement [file jamanetwopen-e2531366-s002.pdf]

Data Sharing Statement

Kim. Antithrombotic Medications and Intraocular Hemorrhage Risk in Exudative Age-Related Macular Degeneration. *JAMA Netw Open*. Published September 11, 2025. doi:10.1001/jamanetworkopen.2025.31366

Data
Data available: No

Additional Information
Explanation for why data not available: The outflow of source data to the outside is strictly prohibited by national security law. The raw data used in this study can be extracted by request from any qualified investigator through the Korean national HIRA system.
